# Supplementary material for: Transcriptional networks specifying homeostatic and inflammatory programs of gene expression in human aortic endothelial cells
Source: eLife. 2017 Jun 6;6:e22536. doi: 10.7554/eLife.22536 (PMC5461113; doi:10.7554/eLife.22536)
Supplement: Figure 6—source data 1. — Differential expression performed in DEseq. DOI: http://dx.doi.org/10.7554/eLife.22536.024 [file elife-22536-fig6-data1.docx]

| **Symbol** | **Entrez Gene Name** | **RefSeq** | **Log2 Ratio (siCEBP/siSCR)** | **FDR** |
| --- | --- | --- | --- | --- |
| IL1B | interleukin 1 beta | NM_000576 | 3.18 | 1.5E-21 |
| IL11 | interleukin 11 | NM_000641 | 2.408 | 4.9E-05 |
| PDIA2 | protein disulfide isomerase family A member 2 | NM_006849 | 2.282 | 1.8E-03 |
| COL25A1 | collagen type XXV alpha 1 | NM_198721 | 2.219 | 1.1E-17 |
| TGFBR1 | transforming growth factor beta receptor 1 | NM_004612 | 2.106 | 3.5E-48 |
| TGFB2 | transforming growth factor beta 2 | NM_001135599 | 2.084 | 1.5E-70 |
| LIF | leukemia inhibitory factor | NM_002309 | 2.015 | 6.7E-16 |
| F2RL1 | F2R like trypsin receptor 1 | NM_005242 | 1.863 | 5.6E-53 |
| IL1A | interleukin 1 alpha | NM_000575 | 1.696 | 2.7E-12 |
| VCAM1 | vascular cell adhesion molecule 1 | NM_001078 | 1.508 | 2.8E-90 |
| PDGFC | platelet derived growth factor C | NR_036641 | 1.306 | 9.8E-12 |
| MYL9 | myosin light chain 9 | NM_006097 | 1.291 | 6.9E-24 |
| ACTA2 | actin, alpha 2, smooth muscle, aorta | NM_001141945 | 1.245 | 2.7E-15 |
| IL1RAP | interleukin 1 receptor accessory protein | NM_001167928 | 1.216 | 8.0E-08 |
| SMAD9 | SMAD family member 9 | NM_001127217 | 1.205 | 2.4E-12 |
| ACVR2B | activin A receptor type 2B | NM_001106 | 1.199 | 4.8E-08 |
| FLT1 | fms related tyrosine kinase 1 | NM_002019 | 1.187 | 9.1E-42 |
| F3 | coagulation factor III, tissue factor | NM_001993 | 1.153 | 1.1E-04 |
| BMPR1A | bone morphogenetic protein receptor type 1A | NM_004329 | 1.112 | 2.4E-03 |
| SELE | selectin E | NM_000450 | 1.106 | 2.9E-42 |
| PIK3CD | phosphatidylinositol-4,5-bisphosphate 3-kinase catalytic subunit delta | NM_005026 | 1.063 | 1.8E-13 |
| EGFR | epidermal growth factor receptor | NM_005228 | 1.051 | 1.5E-18 |
| CCL2 | C-C motif chemokine ligand 2 | NM_002982 | 1.04 | 2.6E-28 |
| MRAS | muscle RAS oncogene homolog | NM_001085049 | 1.014 | 2.4E-06 |
| VEGFC | vascular endothelial growth factor C | NM_005429 | 1.013 | 2.5E-08 |
| IGF1R | insulin like growth factor 1 receptor | NM_000875 | 1.01 | 8.3E-40 |
| NKX2-5 | NK2 homeobox 5 | NM_001166175 | 1.008 | 2.2E-03 |
| RPS6KA6 | ribosomal protein S6 kinase A6 | NM_014496 | 1.007 | 2.8E-02 |
| ITGAV | integrin subunit alpha V | NM_002210 | 1.006 | 2.2E-54 |
| SMAD7 | SMAD family member 7 | NM_005904 | 1.005 | 6.2E-11 |

**Figure 6 - source data 1 -** Transcripts up-regulated by more than 2-fold by CEBPD

knockdown in untreated HAECs compared to scrambled control. Differential expression performed in DEseq.
