# Supplementary material for: Transcriptional networks specifying homeostatic and inflammatory programs of gene expression in human aortic endothelial cells
Source: eLife. 2017 Jun 6;6:e22536. doi: 10.7554/eLife.22536 (PMC5461113; doi:10.7554/eLife.22536)
Supplement: Supplementary file 3. — DOI: http://dx.doi.org/10.7554/eLife.22536.028 [file elife-22536-supp3.docx]

**Supplementary File 3 – List of primers and siRNA oligos used in study**

**Figure 4:**

- (c) siRNAs used:
  - Non-targeting siRNA #4 (siSCR)- Dharmacon #D-001210-04-05
  - siERG Pool (siERG) – Dharmacon #M-003886-01

**Figure 5:**

- (e) qPCR primers used:
  - CEBPb v1 F - ACCCACGTGTAACTGTCAGC
  - CEBPb v1 R – TGCCCCCAAAAGGCTTTGTA
  - CEBPd v2 F – CATCGACTTCAGCGCCTACA
  - CEBPd v2 R – GATTGCTGTTGAAGAGGTCG
  - IRF1 v2 F – GTGAGCGCCTTGGTATGACT
  - IRF v2 R – AGTCAAGTTCAGGCGGGATG
  - GAPDH F - CGACGCCTGCTTCACCTTCTTG
  - GAPDH R - GTCATCATCTCTGCCCCCTCTGCT

**Figure 6:**

- (b, d) siRNAs used:
  - Non-targeting siRNA #4 (siSCR)- Dharmacon #D-001210-04-05
  - siERG Pool (siERG) – Dharmacon #M-003886-01
  - siCEBPD – Dharmacon #M-010453-02
  - siIRF1 – Dharmacon #M-011704-01

**Supplementary Figure 9:**

- Non-targeting siRNA #4 (siSCR)- Dharmacon #D-001210-04-05 (labeled in Figure as siSCR#1)
- Allstars Negative Control – Qiagen SI03650318 (labeled in Figure as siSCR#2)
- JUN Pool – Dharmacon #M-003268-03
- JUND Pool – Dharmacon #M-003900-05
- ERG Pool – Dharmacon #M-003886-01
- ERG #1 – Dharmacon #D-003886-01 (labeled in Figure as siERG#1)
- ERG #2 – Dharmacon #D-003886-02 (labeled in Figure as siERG#2)
- ERG #4 – Dharmacon #D-003886-04 (labeled in Figure as siERG#3)
- ERG #5 – Dharmacon #D-003886-05 (labeled in Figure as siERG#4)
- Hs ERG 3 – Qiagen S100063903 (labeled in Figure as siERG#5)
- Hs ERG 4 – Qiagen S100063910 (labeled in Figure as siERG#6)

**qPCR Primers:**

- JUN-F - GTGCCGAAAAAGGAAGCTGG
- JUN-R – CTGCGTTAGCATGAGTTGGC
- JUNB v1 F – CCGACGACCACCATCAGCTA
- JUNB v1 R – ACTTTGATGCGCTCTTGGTC
- ERG v1 F – CCTAGCCAGGTGAATGGCTC
- ERG v1 R- ATAACTCTGCGCTCGTTCGT
- IL6-F – TCTCCACAAGCGCCTTCGGTCCA
- IL6-R – AGGGCTGAGATGCCGTCGAGGATGTA
- hIL8-F – TCTCTTGGCAGCCTTCCTGATTTC
- hIL8-R – GTGTGGTCCACTCTCAATCAC
- IL1a v1 F – AGTAGCAACCAACGGGAAGG
- IL1a v1 R – AAGGTGCTGACCTAGGCTTG
- IL1b v3 F – CAAAGGCGGCCAGGATATAA
- IL1b v3 R – AAGTGAGTAGGAGAGGTGAGAG
- CCL2 v2 F – TTCCCCTAGCTTTCCCCAGA
- CCL2 v2 R – TCCCAGGGGTAGAACTGTGG
- F3 v1 F – AGTAGCTCCAACAGTGCTTCC
- F3 v1 R – TCATCCTTGTCATCATCCTGGC
- LIF v1 F – GGGGCACCTTCTTCTAGCTC
- LIF v1 R – AACCGAGGGATCTCAGGAGT
- PECAM v1 F – AGACGTGCAGTACACGGAAG
- PECAM v1 R – AGTATCTGCTTTCCACGGCA
- EGFL7 v2 F – TCAGCTGAGGGAAGGTACGA
- EGFL7 v2 R – TTATTGTGCTGGGGGTCAGG
- VWF v1 F – CCATCGAGGTGAAGCACAGT
- VWF v1 R – CCATGTTCCCACCCACGTAA
- CSF3 F - GTTCCCCATCTGGGTCCTTG
- CSF3 R – CGGTGATGTTCGGGAGTCAA
- NOS3 F - TGGCTTTCCCTTCCAGTTCC
- NOS3 R – AATCCTTGCAGCCTCTGGAC
- GAPDH F - CGACGCCTGCTTCACCTTCTTG
- GAPDH R - GTCATCATCTCTGCCCCCTCTGCT
